# *Bartonella* spp. Infections Identified by Molecular Methods, United States

**DOI:** 10.3201/eid2903.221223

**Published:** 2023-03

**Authors:** David W. McCormick, Sara L. Rassoulian-Barrett, Daniel R. Hoogestraat, Stephen J. Salipante, Dhruba SenGupta, Elizabeth A. Dietrich, Brad T. Cookson, Grace E. Marx, Joshua A. Lieberman

**Affiliations:** Centers for Disease Control and Prevention, Fort Collins, Colorado, USA (D.W. McCormick, E.A. Dietrich, G.E. Marx);; University of Washington, Seattle, Washington, USA (S.L. Rassoulian-Barrett, D.R. Hoogestraat, S.J. Salipante, D. SenGupta, B.T. Cookson, J.A. Lieberman)

**Keywords:** *Bartonella*, bacteria, parasites, vector-borne infections, zoonoses, endocarditis, lymphadenitis, cat-scratch disease, polymerase chain reaction, United States

## Abstract

Molecular methods can enable rapid identification of *Bartonella* spp. infections, which are difficult to diagnose by using culture or serology. We analyzed clinical test results of PCR that targeted bacterial 16S rRNA hypervariable V1–V2 regions only or in parallel with PCR of *Bartonella*-specific *ribC* gene. We identified 430 clinical specimens infected with *Bartonella* spp. from 420 patients in the United States. Median patient age was 37 (range 1–79) years; 62% were male. We identified *B. henselae* in 77%, *B. quintana* in 13%, *B. clarridgeiae* in 1%, *B. vinsonii* in 1%, and *B. washoensis* in 1% of specimens. *B. quintana* was detected in 83% of cardiac specimens; *B. henselae* was detected in 34% of lymph node specimens. We detected novel or uncommon *Bartonella* spp. in 9 patients. Molecular diagnostic testing can identify *Bartonella* spp. infections, including uncommon and undescribed species, and might be particularly useful for patients who have culture-negative endocarditis or lymphadenitis.

## Abstract 2

Molecular diagnostic testing can identify these pathogens, including uncommon and previously undescribed species.

*Bartonella* spp. are fastidious, gram-negative intracellular bacteria that are transmitted to humans by insect vectors. The genus includes 12 species associated with human infection: *B. henselae*, *B. quintana*, *B. bacilliformis*, *B. elizabethae*, *B. vinsonii*, *B. koehlerae*, *B. clarridgieae*, *B. alsatica*, *B. doshiae*, *B. grahamii*, *B. rattimassiliensis*, and *B. tribocorum* (*1*,*2*). Bartonellosis cases are not nationally notifiable in the United States, limiting knowledge of disease epidemiology.

In the United States, *B. henselae* is the most common pathogenic *Bartonella* spp.; ≈12,500 cases of infection and 500 hospitalizations occur annually (*3*). The most common clinical manifestation of *B. henselae* infection is cat-scratch disease (lymphadenopathy and fever after a cat scratch or bite), although infection can also cause hepatic lesions, ocular disease, osteomyelitis, and endocarditis (*4*,*5*). *B. quintana* infection is uncommon and incidence is unknown. Clinically, *B. quintana* infection can manifest as acute febrile illness or subacute endocarditis. *B. quintana* is transmitted by body lice and causes the most frequently reported vector-borne disease among persons experiencing homelessness (PEH); seroprevalence in the PEH population is 5%–15% (*6*–*8*). Manifestations of other *Bartonella* spp. infections are sporadically described in case reports, often as culture-negative endocarditis.

Serology is the diagnostic tool most frequently used to identify *Bartonella* spp. infections. However, serologic diagnosis is complicated by lack of species-specific results, differences in use and interpretation of serologic assays among laboratories, and cross-reactivity with other pathogens, including *Chlamydia* spp. and *Coxiella burnettii* (*9*–*15*), which can lead to misdiagnosis and insufficient treatment. Bacterial cultures of blood or tissue can establish the diagnosis, but sensitivity of cultures is low because of the fastidious nature of *Bartonella* spp. and might result in underdiagnosis of infection. Combining enrichment culture techniques with molecular methods might increase detection of *Bartonella* spp. in blood or other clinical specimens (*16*).

Molecular detection of bacterial pathogens has emerged as an important clinical tool that can increase diagnostic yield compared with culture alone, particularly for detection of fastidious organisms (*17*). *Bartonella* spp. have been detected by using broad-range PCR-based assays that target conserved binding sites flanking regions of the rRNA gene and have species-specific variations (*18*) or by using organism-specific gene targets, such as *gltA* (*19*) and *ribC* (*20*). We describe the demographic, clinical, and microbiologic characteristics of patients with bartonellosis diagnosed by both broad-range and organism-specific molecular assays at a large clinical reference laboratory in Washington, USA.

## Materials and Methods

### Population

We included information for all patients who had an acceptable clinical specimen submitted to the University of Washington (Seattle, Washington, USA) Molecular Microbiology clinical diagnostic reference laboratory during 2003–2021. Acceptable specimen types were fresh frozen tissue, formalin-fixed paraffin embedded (FFPE) tissues, and body fluids other than blood. To identify *Bartonella* spp., we performed 16S rRNA sequencing by using broad-range PCR primers (PCR-16S) or next-generation sequencing (NGS-16S) or performed a *Bartonella henselae* and *B. quintana* bi-species–targeted PCR (BT-PCR) requested by the ordering clinician. 

We evaluated patient information if the specimen had *Bartonella* spp. as the final species assignment, had sufficient material for testing, and was an acceptable specimen type for the assay performed and if the presence of PCR inhibitors was excluded (inhibitors precluded ruling out *Bartonella* spp.). If a patient had multiple specimens submitted >30 days apart, we included only the information provided with the first positive specimen submission in the analysis. From the specimen submission form, we obtained the patient’s sex and age, US state and healthcare facility where the specimen was submitted, specimen collection date, and specimen description (e.g., anatomic location).

### Ethical Approval

The study was approved by the University of Washington Institutional Review Board (approval no. STUDY00013877). This study was reviewed in accordance with policies and procedures of the Centers for Disease Control and Prevention and was determined to be exempt from Institutional Review Board requirements.

### Molecular Methods for *Bartonella* spp. Identification

The University of Washington Molecular Microbiology laboratory performed all clinical testing pursuant to its high-complexity Clinical Laboratory Improvements Amendments license and College of American Pathologists accreditation. Laboratory-developed processes were validated in accordance with standards set by the Clinical Laboratory Standards Institute and monitored for quality through regular proficiency testing, biannual external inspections by the College of American Pathologists, and incorporation of control reactions as prescribed by the Clinical Laboratory Improvements Amendments.

We performed DNA extraction as previously described for both fresh and FFPE tissue (*21*), except that we also validated and used multiple DNA extraction kits (QIAGEN) in 2021 because of COVID-19-related supply chain disruptions. We performed broad-range PCR amplification of the V1–V2 hypervariable region of the bacterial 16S rRNA gene (PCR-16S) as previously described (*18*). When sequencing results of 16S PCR products suggested mixed DNA templates, we performed amplicon-based NGS of the V1–V2 16S rRNA locus (NGS-16S) reflexively by using an Illumina Miseq instrument and 250 bp paired-end reads (*22*,*23*). Targeted detection of *Bartonella* spp. by BT-PCR incorporated broad-range 16S primers and 2 primer sets that amplified *ribC* alleles of *B. quintana* and *B. henselae*. The 16S primers also detect other *Bartonella* spp. but at a higher limit of detection: 100 templates per reaction for other *Bartonella* spp. compared with 5–25 for the *Bartonella* species-specific primers. We sequenced PCR products by using the Sanger method and assigned taxonomic classification after BLAST analysis (*24*) by using both the National Center for Biotechnology Information public databases and a curated database containing type strains and RefSeq (https://www.ncbi.nlm.nih.gov/refseq) records (*21*,*22*). Each case was reviewed by 2 independent certified medical laboratory scientists and a board-certified pathologist (*21*).

### Description of Detected *Bartonella* spp.

When available, results from BT-PCR provided specific taxonomic classification of *Bartonella* spp. If BT-PCR results were unavailable, we used results from PCR-16S or NGS-16S. Some 16S rRNA gene V1–V2 sequences lacked sufficient specificity to provide a species-rank classification, either because of close homology to multiple species or lack of homology to any established species. Therefore, we retrospectively analyzed those sequences reported as *Bartonella* spp. by using BLAST to attempt a species-rank classification (defined as 99.7% sequence homology of the V1–V2 sequence). For diagnostic reporting purposes, species with validly published names according to the taxonomic code were identified at the species level in the clinical diagnostic report; otherwise, we identified the genus in accordance with the laboratory’s standard operating procedures (Appendix) (*22*,*23*).

### Statistical Methods

We used χ^2^ tests to compare categorical variables and Mann-Whitney U tests to compare age distributions. For comparisons between patients who had *B. quintana* infections and those who had *B. henselae* infections, we excluded information from patients who had specimen results indicating another *Bartonella* sp. We performed all statistical analyses by using R software (*25*).

To examine the proportion of specimens infected with *Bartonella* spp. by year and US state, we only included information for patients who had specimens submitted for BT-PCR. We excluded specimens submitted for PCR-16S that was used to evaluate a diverse array of bacterial pathogens. To calculate percent positivity of BT-PCR results, we collected data on ordering location (US state) and year of specimen submission for all BT-PCR assays, including those in which *Bartonella* spp. was not detected. Similar denominator data for PCR-16S and NGS-16S testing were not available.

## Results

**Figure 1:**
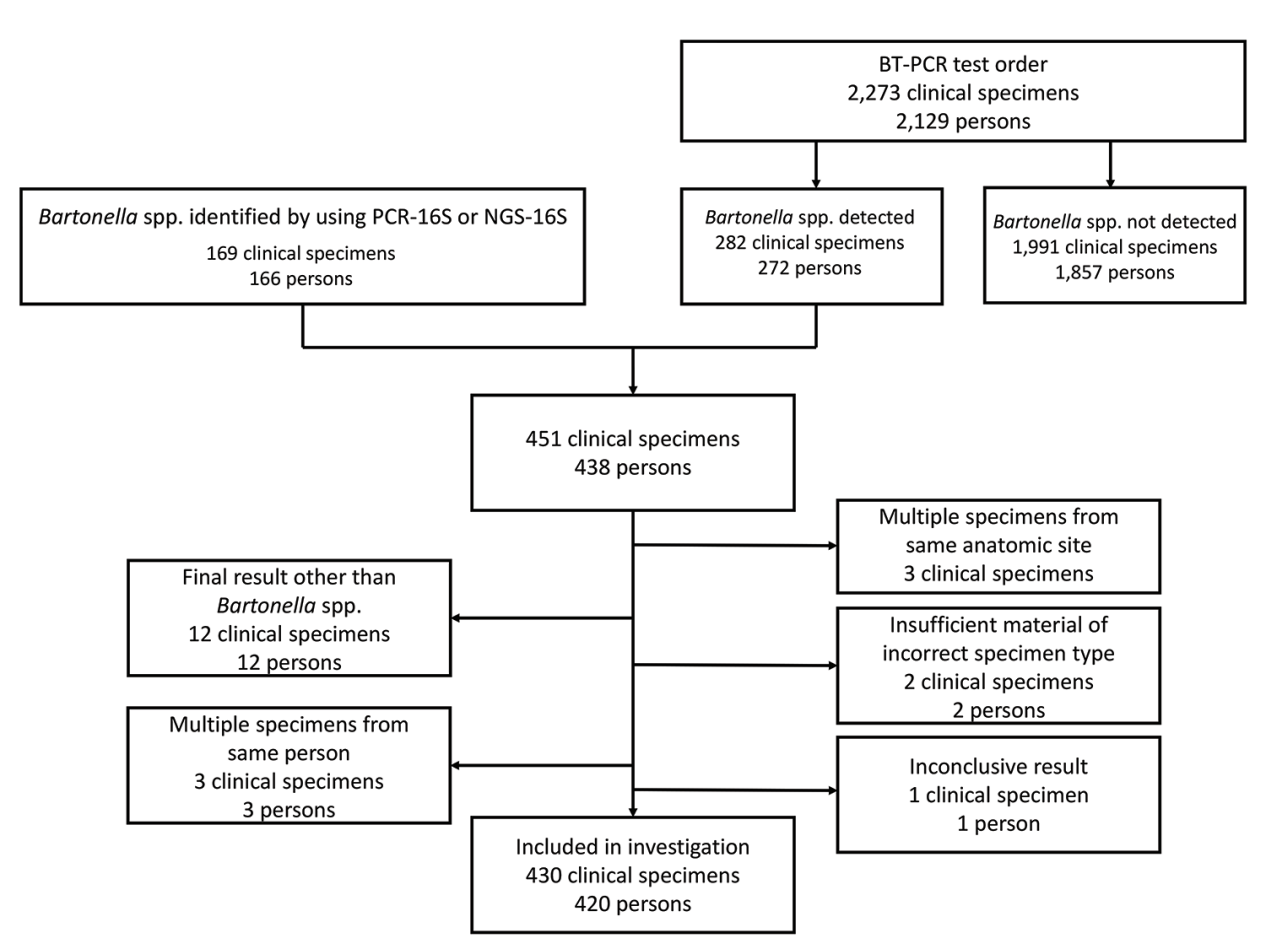
Flow diagram showing clinical specimens included in the analysis in study of *Bartonella* spp. infections identified by molecular methods during 2003–2021 at an academic laboratory in the United States. If a patient had multiple specimens submitted >30 days apart, only information from the first *Bartonella*-positive specimen was included. Clinical specimens were tested for *Bartonella* spp. by PCR. A total of 430 specimens from 420 patients were included in the study. BT-PCR, *B*. *henselae* and *B. quintana* bispecific targeted PCR; NGS-16S, next-generation sequencing of 16S rRNA amplicons; PCR-16S, PCR of 16S rRNA gene followed by Sanger sequencing–based species identification.

We identified 430 clinical specimens from 420 patients that had molecular evidence of *Bartonella* spp. infection (Figure 1). We performed molecular testing by using BT-PCR alone on specimens from 149 (35%) patients, PCR-16S alone (no BT-PCR or NGS-16S) on specimens from 143 (34%) patients, reflexive NGS-16S on specimens from 5 (1%) patients for whom PCR-16S detected multiple bacterial DNA templates (precluding identification of *Bartonella* spp.), and both BT-PCR and PCR-16S sequencing on specimens from 121 (28%) patients. We detected *Bartonella* spp. DNA by both BT-PCR and NGS-16S in a specimen from 1 (<1%) patient and by BT-PCR, PCR-16S, and NGS-16S in a specimen from 1 (<1%) patient. We performed species-level identification for 272/272 (100%) specimens on which we performed BT-PCR, 163/265 (62%) specimens on which we performed PCR-16S sequencing, and 5/7 (71%) specimens on which we performed NGS-16S sequencing. Of 2,273 specimens submitted for BT-PCR during 2003–2021, we identified *Bartonella* spp. in 282 (12%) specimens from 272 patients.

**Figure 2:**
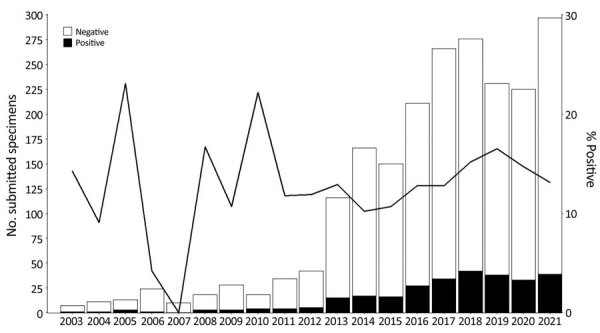
Number and percentage of specimens tested by using *Bartonella-*specific PCR during 2003–2021 in study of *Bartonella* spp. infections identified by molecular methods, United States. A total of 2,273 specimens were submitted for *B*. *henselae* and *B. quintana* bispecific targeted PCR. Bars indicate total numbers of submitted specimens each year and numbers of *Bartonella*-positive or negative specimens. Line indicates percentage of specimens that were positive for *Bartonella* spp.

We identified *Bartonella* spp. in a higher proportion of specimens submitted as unfixed tissue (187/1,276, 16%) than those submitted as FFPE tissue (97/997, 10%; p = 0.0002). The total number of annual specimens submitted for BT-PCR testing increased over time; an annual median of 18 specimens during 2003–2012 increased to an annual median of 225 during 2013–2021 (Figure 2). Overall, we identified *Bartonella* spp. in 13% (260/1,938) of specimens during 2013–2021; peak detection during this period occurred in 2019, when *Bartonella* spp. were detected in 17% of submitted specimens.

Among the 420 patients with detectable *Bartonella* spp., we identified *B. henselae* in specimens from 338 (80%) patients, *B. quintana* from 54 (13%), *B. clarridgeiae* from 4 (1%), *B. vinsonii* from 2 (1%), and *B. washoensis* (GenBank accession no. ON402466) from 1 (1%). We identified *Bartonella* at the genus level in the remaining 21 (5%) patients; of those, we identified 2 candidate species through subsequent analysis: 1 case of endocarditis caused by *Candidatus* Bartonella mayotimonensis (*19*) (GenBank accession no. ON402516) and a unique 16S sequence (tissue specimen; anatomic site not specified by ordering provider) which might represent a previously undescribed *Bartonella* sp. (GenBank accession no. ON402515) (Appendix Table). Of the 420 patients, 411 (98%) had a single submitted clinical specimen. Among the 9 patients with multiple specimens, only 1 *Bartonella* sp. was identified in each patient.

Among 397 patients who had *Bartonella* spp. detected in >1 specimen and available data regarding their sex, 245 (62%) were male (Table 1). A higher proportion of patients who had detectable *B. quintana* (41/50, 82%) were male compared with those who had detectable *B. henselae* (187/321, 58%; p = 0.001). Age at the time of specimen collection was available for 415/420 (99%) patients; median age was 37 years (range 1–79 years). Most (235/415, 57%) patients were 18–65 years of age, 134/415 (32%) were <18 years of age, and 45/415 (11%) were >65 years of age. Patients who had detectable *B. quintana* in >1 specimen (median age 52 years, interquartile range [IQR] 45–60 years) were older than those who had detectable *B. henselae* (median age 32 years, IQR 11–54 years; p<0.0001).

**Table 1:** Demographic characteristics and specimen origin for patients who had *Bartonella* spp. detected by PCR in study of Bartonella spp. infections identified by molecular methods, United States*

Variable	No. patients†	*B*. *henselae*, n = 338	*B*. *quintana*, n = 54	Other *Bartonella* spp.,‡ n = 28
Age, y, median (range)	415	32 (1–79)	52 (9–76)	30 (1–77)
<18	NA	122/335 (36)	2/52 (4)	10/28 (36)
18–65	NA	182/335 (54)	43/52 (83)	11/28 (39)
>65	NA	31/335 (9)	7/52 (13)	7/28 (25)
Sex	397	NA	NA	NA
M	NA	187/321 (58)	41/50 (82)	17/26 (65)
F	NA	134/321 (42)	9/50 (18)	9/26 (35)
Specimen origin§	361	NA	NA	NA
Texas	48	38/48 (79)	4/48 (8)	6/48 (13)
Washington	46	34/46 (74)	8/46 (17)	4/46 (9)
Ohio	40	36/40 (90)	0/40 (0)	4/40 (10)
California	40	22/40 (55)	16/40 (40)	2/40 (5)
Michigan	30	27/30 (90)	3/30 (10)	0/30 (0)
Florida	27	26/27 (96)	0/27 (0)	1/27 (4)
Oregon	21	17/21 (80)	2/21 (10)	2/21 (10)
Pennsylvania	11	10/11 (91)	0/11 (0)	1/11 (9)
Other¶	NA	78/98 (80)	15/98 (15)	5/98 (5)

*Values are no./total no. (%) unless otherwise indicated. NA, not applicable. †Total number of patients for each indicated variable. ‡Includes *B. vinsonii* (n = 2), *B*. *clarridgeiae* (n = 4), *B. washoensis* (n = 1), and *Bartonella* sp. not otherwise specified (n = 21). §Restricted to states with >10 patients who were infected with an identified *Bartonella* spp. to preserve anonymity. ¶Alaska (n = 4), Alabama (n = 5), Arkansas (n = 5), Colorado (n = 5), Connecticut (n = 3), District of Columbia (n = 2), Georgia (n = 5), Hawaii (n = 2), Iowa (n = 3), Idaho (n = 1), Illinois (n = 3), Indiana (n = 6), Kentucky (n = 2), Louisiana (n = 1), Massachusetts (n = 5), Maine (n = 1), Montana (n = 5), Mississippi (n = 1), North Carolina (n = 8), Nebraska (n = 5), New Hampshire (n = 2), New Jersey (n = 1), New York (n = 3), Oklahoma (n = 1), South Carolina (n = 3), Tennessee (n = 5), Utah (n = 1), Virginia (n = 5), and Wisconsin (n = 5).

**Figure 3:**
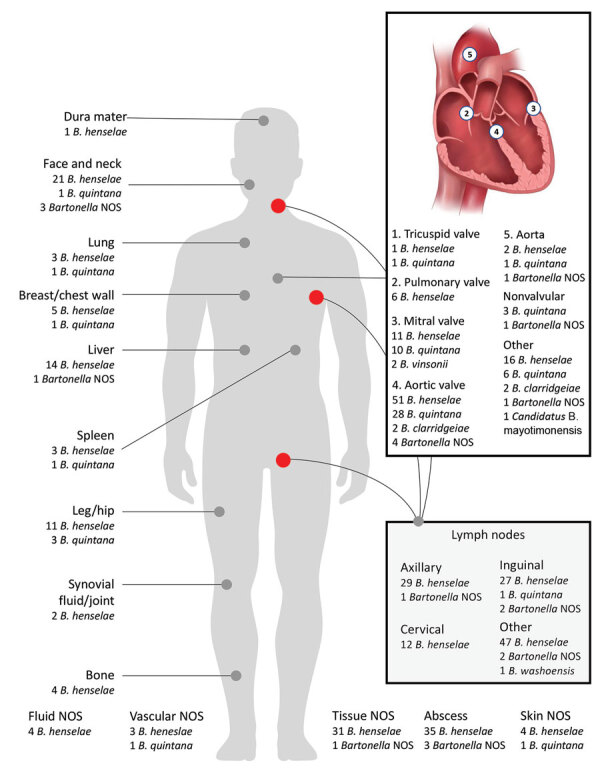
Frequency of *Bartonella* spp. from different anatomic sites identified during 2003–2021 in study of *Bartonella* spp. infections identified by molecular methods, United States. Multiple specimens were submitted for 9 patients. We detected *Bartonella* spp. in both splenic and cardiac specimens from 1 patient, in 2 cardiac specimens each from 7 patients, and in 3 cardiac specimens from 1 patient. If we detected *Bartonella* spp. on multiple valve specimens, those were included in the total count for all involved valves. For the heart valve inset, other sites are cardiac tissue NOS (n = 18), right ventricular outflow tract conduit (n = 3), pacemaker or implantable cardiac device lead (n = 4), and coronary cusp (n = 1). For the lymph node inset, other sites are lymph node NOS (n = 38), supraclavicular (n = 3), submental (n = 2), mesenteric (n = 1), preauricular (n = 1), submandibular (n = 1), epitrochlear (n = 1), jugular (n = 1), iliac (n = 1), and paraspinal (n = 1). NOS, not otherwise specified.

Specimen descriptions were provided for all specimens. The most common specimens submitted were cardiac (150/430, 35%), lymph node biopsy or aspirate (122/430, 29%), and abscess fluid (38/430, 9%) (Figure 3). We identified *B. quintana* more frequently in cardiac specimens (45/54, 83%) and *B. henselae* more frequently in lymph node specimens (115/338, 34%). We detected *B. henselae* in axillary (29/115, 25%), inguinal (27/115, 23%), and cervical (12/115, 10%) lymph node specimens and in cardiac (82/338, 24%), abscess (35/338, 10%), and liver (14/338, 4%) specimens. We detected *B. washoensis* in 1 lymph node specimen (unspecified anatomic location) and *B. vinsonii* (n = 2) and *B. clarridgeiae* (n = 4) only in cardiac specimens. For patients with *Bartonella* spp. identified in cardiac specimens, the aortic valve (85/140, 61%) was most frequently involved, followed by the mitral valve (23/140, 16%). We detected *Bartonella* spp. in >1 valve from 8 (6%) patients. Of cardiac specimens that had >1 valve affected, 105/116 (90%) were from the left side of the heart.

We observed a higher proportion of *Bartonella* spp. in cardiac tissue of male (107/245, 44%) than female (24/152, 16%) patients. However, 57 (38%) female patients had *Bartonella* spp. detected in lymph node specimens compared with 61 (25%) male patients, and 71 (47%) female patients had *Bartonella* spp. detected in other (not cardiac or lymph node) specimen types, compared with 77 (31%) male patients (p<0.0001) (Table 2). For patients <18 years of age, we detected *Bartonella* spp. more frequently in lymph node (52/134, 39%) or other noncardiac specimen types (77/134, 57%), whereas *Bartonella* spp. was most frequently identified in cardiac specimens from patients who were 18–65 (106/235, 45%) and >65 (27/45, 60%) years old (p<0.0001). For patients <18 years of age, *B. henselae* was the most commonly identified species (122/134, 91%).

**Table 2:** Categorical age and sex of infected persons grouped by specimen type in study of *Bartonella* spp. infections identified by molecular methods, United States

Variable	No. (%) specimens			p value†
	Cardiac, n = 140	Lymph node, n = 122	Other,* n = 158	
Age, y				<0.0001
<18, n = 134	5 (4)	52 (39)	77 (57)	
18–65, n = 236	106 (45)	66 (27)	64 (27)	
≥65, n = 45	27 (60)	3 (7)	15 (33)	
Sex				<0.0001
M, n = 245)	107 (44)	61 (25)	77 (31)	
F, n = 152)	24 (16)	57 (38)	71 (47)	

*Abscess (n = 38), tissue (n = 32), neck/face/arm (n = 25), liver (n = 15), leg/hip (n = 14), breast/chest wall (n = 6), skin (n = 5), lung (n = 4), aorta (n = 2), vascular (n = 2), body fluid NOS (n = 4), bone (n = 4), spleen (n = 4), synovial fluid (n = 2), and dura mater (n = 1). †By χ^2^ test.

Information on the origin of specimens was available for 361/420 (86%) patients, representing 36 states and the District of Columbia (Table 1). The states providing the greatest number of specimens were Texas (48/361, 13%) and Washington (46/361, 13%); specimens were provided for >10 patients in 8 (22%) states and for <5 patients in 16 states and the District of Columbia (46%). Among states with >10 specimens in which we identified *Bartonella* spp., California (16/40, 40%) and Washington (8/46, 17%) had the highest proportion of specimens infected with *B. quintana*; *B. quintana* was not identified in >10% of specimens in any other state.

## Discussion

We report 420 cases of bartonellosis in the United States identified by uniform, clinical molecular diagnostic methods at a single reference laboratory during 2003–2021, representing 36 US states and including 140 persons who had endocarditis because of *Bartonella* spp. infections. Those cases highlight the broad clinical spectrum of disease caused by *Bartonella* spp., provide evidence that multiple species can cause bartonellosis, and demonstrate the utility of clinical molecular testing for pathogen identification.

*B. henselae* was the most frequently identified species causing bartonellosis. Although *B. henselae* was most often detected in lymph node specimens, consistent with cat-scratch disease (*3*), this species was also identified in cardiac specimens, underscoring its potential to cause endocarditis and endovascular disease (*26*). *B. henselae* was also detected in a diverse range of clinical specimens, including liver and bone, indicating the breadth of atypical *B. henselae* infections (*4*,*5*). Although atypical bartonellosis manifestations are rare, ≈25% of pediatric hospitalizations associated with cat-scratch disease are caused by atypical *B. henselae* infections (*27*). A previous study of US insurance claims data found that atypical infections with *B. henselae* accounted for 1.5% of cases; ocular and hepatic lesions were the most common clinical manifestations of atypical *B. henselae* infection (*28*). Overall, *Bartonella* spp. were more frequently found in cardiac specimens in our study, which might reflect the preponderance of certain specimen types submitted for molecular diagnostic tests.

*B. quintana* was the second most frequently identified species overall and in cardiac specimens, which is consistent with *B. quintana* as a causative agent of subacute endocarditis (*11*,*26*,*29*). California and Washington had the highest proportion of specimens infected with *B. quintana*; a high prevalence of antibodies against *B. quintana* has been described among PEH living in both states (*6*,*7*). Eight states reported >10 cases of bartonellosis during the study period, and Texas and Washington reported the highest number of cases. The higher proportion of *B. quintana* infections observed in California and Washington might be from epidemiologic clustering, because *B. quintana* is transmitted by body lice, which can be spread through shared clothing and bedding material, and clusters of *B. quintana* infections have been reported among PEH (*6*,*8*,*30*). The higher number of *B. quintana* infections in California and Washington might reflect geographic differences in the number or type of specimens submitted for molecular testing. Although other studies have reported that *B. henselae* infection is more common in the southeastern region of the United States (*3*,*28*), we did not identify a clear predominance of infections in this region, which might reflect specimen submission patterns. Despite a potential bias from specimen submission patterns, molecular testing methods might identify spatiotemporal clusters of *B. quintana* infections.

Persons who had *B. henselae* infections were younger, and a higher proportion were female compared with those who had *B. quintana* infections. This finding likely reflects the higher incidence of *B. henselae* infection in children, possibly because children might spend more time in close proximity with domesticated cats (*3*). Persons <18 years of age commonly had lymph node or abscess involvement, typical of cat-scratch disease (*5*).

Nearly all patients with endocarditis had left-sided disease, which is consistent with other reports of endocarditis caused by *Bartonella* spp. (*11*,*29*,*31*). We found that *B. henselae* was a more frequent cause of endocarditis in our study than in prior studies that used molecular methods (*26*,*29*). In a study of 685 patients in the United Kingdom who had endocarditis, *B. quintana* was identified in 12/13 cases that had available PCR diagnostic results; *B. henselae* was identified in only 1/13 cases (*29*). A retrospective review of cases using PCR testing on heart valves identified *B. quintana* in 26/45 patients, compared with 19/45 patients who had *B. henselae* infections (*26*). However, the differences observed in our study might be because of differences in sample submission practices; we did not have information from the referring hospitals on the total number of patients with endocarditis caused by *Bartonella* spp. Although previous reviews have recommended the use of serologic testing to diagnose endocarditis caused by *Bartonella* spp. (*31*), our findings, in combination with those of other large studies (*26*,*29*), underscore the improved accuracy of molecular testing methods in obtaining a definitive diagnosis.

We identified 9 patients who had infections from novel or newly emerging *Bartonella* spp., including 2 patients with endocarditis caused by *B. vinsonii* and 4 patients with endocarditis caused by *B. clarridgieae*. Both *B. vinsonii* and *B. clarridgieae* have been reported to cause endocarditis (*20*,*26*,*32*), although only 1 case of endocarditis caused by *B. clarridgieae* infection has been previously reported (*32*); that infection was also confirmed by using molecular methods. We detected *Candidatus* B. mayotimonensis in 1 case of endocarditis, similar to findings for a single case reported previously (*19*). We also detected a case of *B. washoensis* lymphadenitis; this bacterium has been detected in ground squirrels and their fleas and was reported to be the etiologic agent in 2 human cases of myocarditis and meningitis in the United States (*33*) and prosthetic valve endocarditis in a patient in Germany (*34*). We detected a novel 16S rRNA sequence variant of *Bartonella*, most likely representing a previously undescribed species. 

Although serology is an important diagnostic method for bartonellosis, case reports have described cross-reactivity of antibodies against *B. clarridgieae*, *B. henselae*, and *B. quintana* (*32*,*35*,*36*) and cross-reactivity between antibodies against *B. vinsonii* and *Coxiella burnetii* (*20*), highlighting the utility of molecular methods for species-specific diagnosis of those pathogens. Because *B. quintana*, *B. henselae*, and other non–*Bartonella* spp. pathogens are often treated with different antimicrobial drugs and for different durations, identification of infecting species can frequently have major effects on treatment, in addition to the epidemiologic value of recognizing novel organisms.

Despite increased specificity of molecular methods compared with serology and potential to detect novel organisms, disparate PCR positivity rates for fresh (16%) and FFPE (8%) tissue specimens highlight how preanalytical factors, such as formalin fixation, can limit assay yield. Other potential factors that can limit sensitivity of molecular methods include specimen selection, organism prevalence, and tissue volume. Formalin fixation damages DNA and can reduce assay sensitivity but has not always reduced positivity rates, perhaps because histopathologic evaluation permits selection of tissue most likely to contain microbial DNA (*21*). In this study, lymph nodes constituted the highest proportion of FFPE specimens. The observed discordant PCR positivity between specimen types might reflect anatomic site of disease, volume of tissue available for PCR, or tissue-specific variations in organism prevalence.

The first limitation of our study is that the analyzed specimens were not representative of all infections caused by *Bartonella* spp. because they represented more severe illness, and submitted specimens were primarily from tissues known to be infected by those pathogens. Second, the specimens did not represent a random sample of persons with bartonellosis; therefore, we could not estimate incidence or perform statistical inference testing. Third, not all geographic regions or states were equally represented, limiting our ability to compare results between different geographic areas. Fourth, we had little information regarding clinical features of the patients from whom specimens were collected, limiting our ability to determine potential risk factors on the basis of medical or social history. Fifth, preanalytical specimen handling can affect diagnostic yield; we could not assess or control variations in storage or transport conditions or tissue processing, such as formalin pH or acid-based decalcification of bone. Sixth, clinical testing might have missed *Bartonella* spp. other than *B. henselae* or *B. quintana* that were present at levels below the limit of detection for the 16S primers, because the *ribC* primers were designed to increase specificity and sensitivity for detecting *B. henselae* and *B. quintana*. Finally, some specimens might have been misclassified, leading to a higher proportion of specimens categorized as abscesses or tissue if more specific information on anatomic site was not provided on the specimen submission form.

In conclusion, molecular methods provide a powerful diagnostic tool to detect infections caused by *Bartonella* spp. Those methods should be considered for patients who have culture-negative endocarditis or lymphadenitis of unclear etiology, particularly in persons with established risk factors, including exposure to cats or prior homelessness. Broader use of molecular methods in suspected cases of bartonellosis will help elucidate the full clinical spectrum of *Bartonella* infections and increase awareness of this underrecognized pathogen.

Appendix Additional information for *Bartonella* spp. infections identified by molecular methods, United States.
