# Supplementary material for: Bartonella spp. Infections Identified by Molecular Methods, United States
Source: Emerg Infect Dis. 2023 Mar;29(3):467–76. doi: 10.3201/eid2903.221223 (PMC9973681; doi:10.3201/eid2903.221223)
Supplement: Appendix — Additional information for Bartonella spp. infections identified by molecular methods, United States. [file 22-1223-Techapp-s1.pdf]

Article DOI: https://doi.org/10.3201/eid2903.221223

EID cannot ensure accessibility for supplementary materials supplied by authors. Readers who have difficulty accessing supplementary content should contact the authors for assistance.

Bartonella spp. Infections Identified by Molecular Methods, United States

Appendix

Additional Methods

Diagnostic Reporting Criteria

When reporting results of clinical diagnostic tests, the laboratory standard operating procedure provides for reporting the most specific taxonomic rank possible on the basis of sequencing results, availability of published sequences, and species defined as validly published by the International Code of Nomenclature of Bacteria [Bacteriological Code]. For clinical diagnostic purposes, the laboratory does not report species with *Candidatus* status or those that are effectively published but not validly published under the Bacteriological Code. Detected organisms without a validly published species name may be reported at the genus rank (e.g., *Bartonella* sp.) or as related to a validly published organism (e.g., *Bartonella* sp. related to *B. quintana*).

Appendix Table. Taxonomic identification quality metrics for selected bacteria in study of Bartonella spp. infections identified by molecular methods, USA*

Species	Accession no.†	Trimmed length	Top BLAST hits		% Identity (alignment length)	Other top BLAST hits		% Identity (alignment length)
			Species	RefSeq accession no.‡		Species	Accession no.†	
B. washoensis	ON402466	290	*B. washoensis*	NZ_UAQI01000042 (R)	100.0 (290)	*B. jaculi*	AB602528	99.2 (258)
			B. washoensis	NZ_JH725101–3 (R)	100.0 (290)	*Candidatus B. mayotimonensis*	HM636441	98.3 (290)
			B. massiliensis	NZ_CABFVS010000059 (T)	98.6 (290)			
			B. birtlesii	NZ_CM001557 (T)	98.6 (290)			
			B. koehlerae	NR_024932 (T)	98.6 (290)			
			B. grahamii	NR_029366 (T)	98.6 (290)			
			B. taylori	NZ_CADEAG010000019 (R)	98.6 (290)			
Candidatus B. mayotimonensis	ON402516	281	*B. senegalensis*	NZ_HE997546 (T)	99.3 (281)	*Candidatus B. mayotimonensis*	FJ376733	100.0 (281)
			B. quintana	NR_044748 (T)	99.3 (281)	*B. schoenbuchensis*	AJ278188–90	98.6 (281)
			B. birtlesii	NZ_CM001557 (T)	98.9 (282)			
			B. vinsonii	NZ_CADEAE010000020 (T)	98.9 (281)			
			B. grahamii	NR_029366 (T)	98.6 (281)			
			B. massiliensis	NZ_CABFVS010000059 (T)	98.6 (282)			
			B. doshiae	NR_029368 (T)	98.6 (281)			
Bartonella sp.	ON402515	253	*B. vinsonii*	NZ_JH725037 (T)	98.8 (253)	*B. grahamii*	AB426634	99.2 (249)
			B. grahamii	NR_029366 (T)	98.8 (253)	*Candidatus B. mayotimonensis*	FJ376733	98.8 (253)
			B. doshiae	NZ_JAGY01000010 (T)	98.4 (253)	*B. grahamii*	NC_012846	98.8 (253)
			B. birtlesii	NR_025051 (T)	98.4 (253)			
			B. henselae	NC_005956 (T)	98.4 (253)			

*Bartonella spp. sequences from specimens were deposited in GenBank and analyzed by using BLAST (https://blast.ncbi.nlm.nih.gov). Trimmed and alignment lengths represent the number of nucleotides.
†GenBank accession numbers.
‡Accession numbers from GenBank RefSeq sequence database (https://www.ncbi.nlm.nih.gov/refseq). T indicates sequence from type strain material and R indicates non-type strain RefSeq records.
